# Molecular contribution of BRCA1 and BRCA2 to genome instability in breast cancer patients: review of radiosensitivity assays

**DOI:** 10.1186/s12575-020-00133-5

**Published:** 2020-10-01

**Authors:** Fatemeh Sadeghi, Marzieh Asgari, Mojdeh Matloubi, Maral Ranjbar, Nahid Karkhaneh Yousefi, Tahereh Azari, Majid Zaki-Dizaji

**Affiliations:** 1 Department of Immunology, School of Medicine, Iran University of Medical Sciences, Tehran, Iran; 2 Digestive Diseases Research Institute, Digestive Oncology Research Center, Tehran University of Medical Sciences, Tehran, Iran; 3 Rheumatology Research Center, Tehran University of Medical Sciences, Shariati Hospital, Kargar Ave, Tehran, Iran; 4 Cancer Research Center, Cancer Institute of Iran, Tehran University of Medical Sciences, Tehran, Iran; 5 Legal Medicine Research Center, Legal Medicine Organization, Tehran, Iran; 6 Research Center for Immunodeficiencies, Children’s Medical Center, Tehran University of Medical Sciences, Tehran, Iran

**Keywords:** BRCA1, BRCA2, breast cancer, genome stability, radiosensitivity, DNA repair pathway, cell cycle, apoptosis, homologous recombination, non-homologous end joining

## Abstract

**Background:**

DNA repair pathways, cell cycle arrest checkpoints, and cell death induction are present in cells to process DNA damage and prevent genomic instability caused by various extrinsic and intrinsic ionizing factors. Mutations in the genes involved in these pathways enhances the ionizing radiation sensitivity, reduces the individual’s capacity to repair DNA damages, and subsequently increases susceptibility to tumorigenesis.

**Body:**

BRCA1 and BRCA2 are two highly penetrant genes involved in the inherited breast cancer and contribute to different DNA damage pathways and cell cycle and apoptosis cascades. Mutations in these genes have been associated with hypersensitivity and genetic instability as well as manifesting severe radiotherapy complications in breast cancer patients. The genomic instability and DNA repair capacity of breast cancer patients with BRCA1/2 mutations have been analyzed in different studies using a variety of assays, including micronucleus assay, comet assay, chromosomal assay, colony-forming assay, γ -H2AX and 53BP1 biomarkers, and fluorescence in situ hybridization. The majority of studies confirmed the enhanced spontaneous & radiation-induced radiosensitivity of breast cancer patients compared to healthy controls. Using G2 micronucleus assay and G2 chromosomal assay, most studies have reported the lymphocyte of healthy carriers with BRCA1 mutation are hypersensitive to invitro ionizing radiation compared to non-carriers without a history of breast cancer. However, it seems this approach is not likely to be useful to distinguish the BRCA carriers from non-carrier with familial history of breast cancer.

**Conclusion:**

In overall, breast cancer patients are more radiosensitive compared to healthy control; however, inconsistent results exist about the ability of current radiosensitive techniques in screening BRCA1/2 carriers or those susceptible to radiotherapy complications. Therefore, developing further radiosensitivity assay is still warranted to evaluate the DNA repair capacity of individuals with BRCA1/2 mutations and serve as a predictive factor for increased risk of cancer mainly in the relatives of breast cancer patients. Moreover, it can provide more evidence about who is susceptible to manifest severe complication after radiotherapy.

## Introduction

The genomic content of cells is constantly exposed to extrinsic and intrinsic factors leading to DNA damage and genomic instability. These damages can affect the integrity of one or both strands of a DNA molecule. In this case, they are called single-strand and double-strand DNA breaks, respectively [1]. Double-strand breaks (DSBs) are considered as much severe and harmful damages, which can generate extreme and disruptive mutations [2]. Several cascades of cellular events comprising DNA repair pathways, cell cycle arrest, and apoptosis are important to rectify DNA damage, prevent uncontrolled cell dividing and passing unrepair DNA damages to the daughter cells. People with mutations in the genes involved in these pathways are more sensitive to radiation (radiosensitive) and have an impaired proliferative capacity after exposure to DNA damaging agents; therefore, they are at higher risk of cancer development compared to a normal population.

Breast cancer is the most common cancer and the first leading cause of cancer-related death in women worldwide [3]. While majority of breast cancers occur sporadically, approximately 5-10 % of them follow a hereditary pattern, meaning that certain mutated genes that are passed from parents to children contribute to the development of breast cancer [4]. Several studies demonstrated an increased level of radiosensitivity among breast cancer patients. They are more radiosensitive comparing to other cancer types, like oesophageal cancer as well [5]. In this malignancy, BRCA1 and BRCA2 are among the high penetrant susceptibility genes and mutations in these genes have been associated with hypersensitivity and genetic instability [6]. Studies have reported that BRCA1^−/−^ mouse embryonic fibroblasts (MEFs) and human breast cancer line, HCC1937 [7, 8], are highly sensitive to ionizing radiation and retrovirally transfecting these cells with wild-type BRCA1 diminished the ionizing radiation sensitivity and improved the efficiency of DSBs repair [9]. Likewise, the clinical studies stated BRCA1/2 mutation carriers are more radiosensitive than healthy control [5, 10, 11] and manifest more severe radiotherapy complications [12, 13] due to having defective DNA damage repair system.

Currently, the most reliable test for pre-screening the BRCA1/2 carriers is limited to the full sequencing of the genes. However, this technique is time-consuming, difficult, and costly and, up to 30% of mutations cannot be detected properly. Moreover, one-third of all breast cancer occurs within the families are not related to either BRCA1 or BRCA2, indicating that other low penetrate genes are involved in the development of familial breast cancer. Evaluating the DNA repair capacity may serve as a biomarker to identify individuals at increased risk of breast cancer and act as a pre-screening test in women with a family history of breast cancer. To date, several studies have utilized different types of assays to evaluate the radiosensitivity in BRCA1/BRCA2-associated breast cancer patients compared to sporadic one and healthy individuals. Here, we first give an overview of the contribution of BRCA1/2 to radiosensitivity through regulating the DNA repair pathways, and cell cycle checkpoint and apoptosis cascades. We then discuss the clinical and functional assays for determining the radiosensitivity capacity of sporadic and familial breast cancer patients.

## DNA repair pathways and cell cycle mechanisms

DSBs are regarded as severe and harmful damages and can generate extreme and disruptive mutations if remain unrepaired [2]. Cells have developed two main repair pathways, homologous recombinant (HR) and non-homologous end-joining (NHEJ) repair pathway, to deal with this type of DNA damage.

**Fig. 1:**
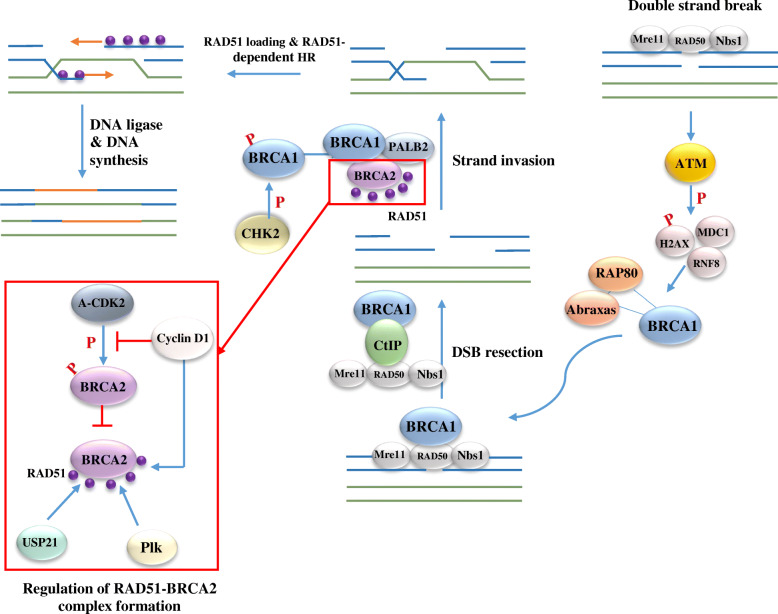
Homologues recombinant DNA repair system. The overall process starts with the recognition of the DSB region by the Mre11-RAD50-Nbs1 (MRN) complex. Next, ATM phosphorylates γH2AX, MDC1, and RNF8, which subsequently initiate the formation of BRCA1–abraxas–RAP80 complex. Later, BRCA1 via cooperating with MRN forms a complex with CtIP, to promote 5′-end resection in the early steps of the synthesis-dependent strand annealing (SDSA) pathway of HR. BRCA1 interacts with PALB2 and BRCA2 to recruit RAD51, an essential mediator in the HR repairing pathway. The formation of BRCA1- PALB2- BRCA2 complex is relying on CHK2-mediated phosphorylation of S988 on BRCA1

HR repair pathway exclusively takes place in the late S and G2 phases of the cell cycle. This pathway requires an unharmed homologous DNA sequence located in the sister chromatin as a template for the synthesis of the damaged region. The overall process starts with the recognition of the DSB region by the Mre11-RAD50-Nbs1 (MRN) complex (Fig. 1). Next, ATM is recruited to the DNA damage location, which in turn facilitates the recruitment of other crucial proteins, including ATR, CHEK2, BARD1, BRCA1, BRCA2, and RAD51 [14]. Mutations in genes encoding these proteins have been associated with the increased risk of breast cancer.

**Fig. 2:**
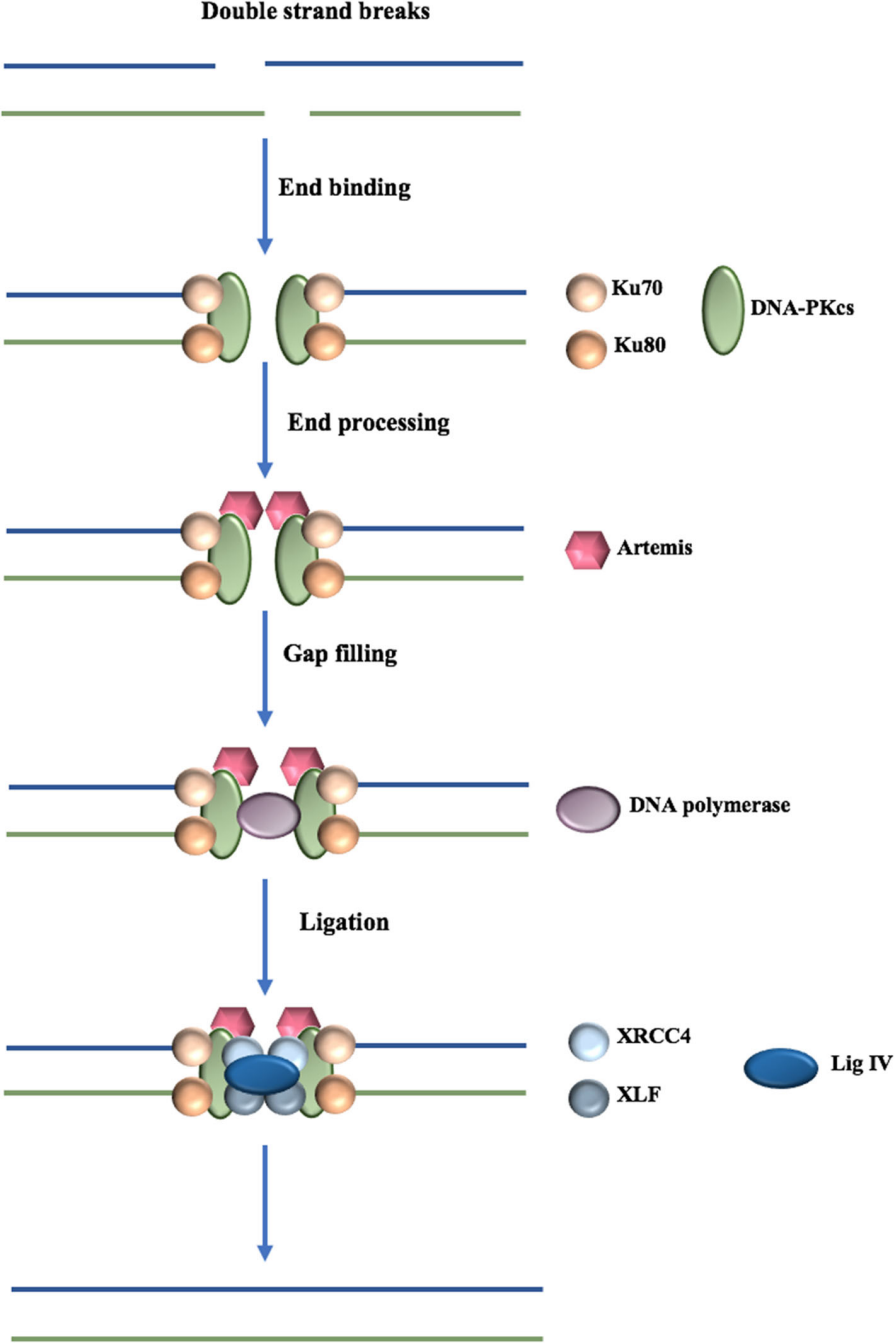
Non-homologous end-joining DNA repair system. The general mechanism involves the recruitment of DNA dependent protein kinases (DNA-PK), following the attachment of Ku proteins on the broken ends of DNA. Afterward, a DNA polymerase fills the gaps that have been produced as a result of the endonuclease activity of Artemis protein. Finally, a DNA ligase IV joins the DNA ends with the help of its cofactors, XRCC4, and XLF

While HR repair is considered as the most accurate and error-free pathway, NHEJ is a less precise repair pathway and is mainly activated in phases G_0_ and G_1,_ where HR is not available. NHEJ also functions as a backup repair pathway in case of defects in components of the HR pathway [15]. The general mechanism involves the recruitment of DNA dependent protein kinases (DNA-PK) [16], following the attachment of Ku proteins on the broken ends of DNA [17] (Fig. 2). Afterward, a DNA polymerase fills the gaps that have been produced as a result of the endonuclease activity of Artemis protein. Finally, a DNA ligase IV joins the DNA ends with the help of its cofactors, XRCC4 and XLF [15].

**Fig. 3:**
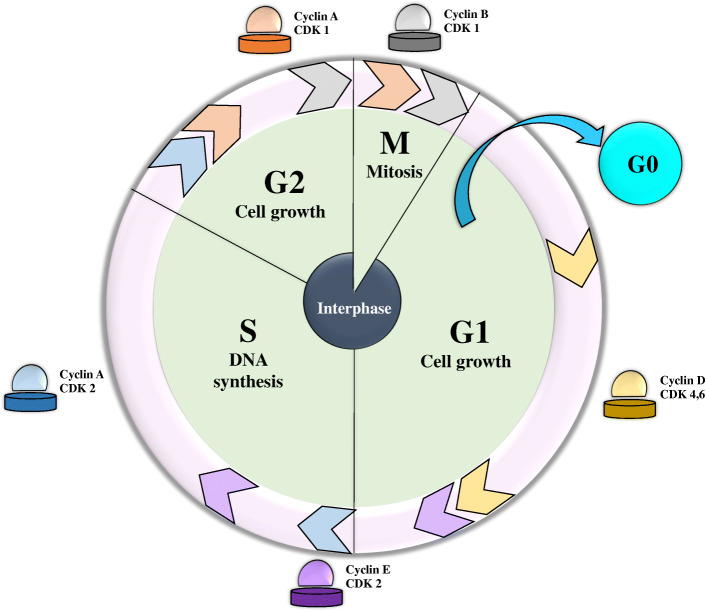
An overview of the cell cycle regulation. Cyclin D with the cooperation of CDK4/6 regulates the events is the early G1 phase. Cyclin E-CDK2 are responsible to initiate S phase, Cyclin A with CDK2 and then CDK1 involve in the completion of S phase for entry into mitosis, and Cyclin B-CDK1 fascinate this entry

Every event that has been described above happens during the cell cycle (Fig. 3). While a normal cell cycle is vital for the development and survival of organisms, a defective one inflicts irreparable losses. To prevent such unwanted destiny, cells have developed cell cycle checkpoints to allow the progression of the cycle when the events of each phase are completed properly or arrest the cycle once there is DNA damage. The main regulators of cell cycle checkpoints are cyclin-dependent kinases (CDK), which are activated in the presence of cyclin proteins [18].

In the G_1_ phase, two complexes of CDK4/6-Cyclin D and CDK2-Cyclin E, permit the cells to enter the S phase by phosphorylating the transcriptional repressors Retinoblastoma (Rb) and p107/p130 proteins, a process which eventually leads to initiation of DNA replication (Fig. 3). In the case of DSB, the cycle is halted by activation of ATM and phosphorylation of CHK2, which in turn phosphorylate cdc25A and p53 in order to inhibit the cell from entering the S phase [19]. Cells that have successfully passed the G_1_ checkpoint, start their DNA duplication in phase S. A DNA damage in this stage leads to activation of the ataxia-telangiectasia mutated and Rad3 related kinase (ATR) and chk1 kinase to stabilize p53 and degrade cdc25A [18, 20].

Prior to the mitosis, cells go through the G_2_ phase to grow and produce the proteins necessary for the division process (Fig. 3). In this phase, CDK1-cyclin B is the main regulator and the interruption of the cell cycle in the presence of DNA damage particularly relies on ATR and chk1 rather than ATM and CHK2 proteins [18].

The activity of each component in DNA repair pathways and cell cycle checkpoints results in the progression of cells into division or apoptosis. However, in cancerous cells, these mechanisms do not function properly and lead to harmful consequences.

## BRCA1

### Frequency

Mutation in BRCA1 gene is considered as the main cause of hereditary breast cancer, and it is responsible for 40–45% of total hereditary breast cancer development [21]. Over 858 BRCA1 mutations have been confirmed to have a significant clinical impact on cancer susceptibility. Women with an inherited BRCA1 mutation have a lifetime risk of 70–80% of developing breast cancer and 37–62% of developing ovarian cancer [22]. Moreover, there are other types of cancers related to the BRCA1 mutations, such as fallopian tube and peritoneal cancer in women and prostate and breast cancer in men [23, 24].

BRCA1 mutated breast cancer is known to be triple-negative breast cancers (TNBC), characterized by negative estrogen receptor (ER), progesterone receptor (PR), and human epidermal growth factor receptor 2 (HER2). However, they manifest the same immunohistochemical profiles for the positive expression of cytokeratin (CK) 5/6 and CK14 with sporadic basal carcinoma [25].

### Gene structure

**Fig. 4:**
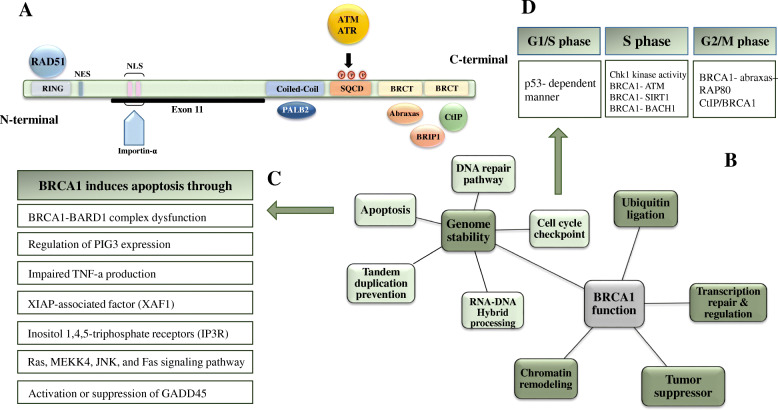
The gene structure of BRCA1 (**a**) and the overall contribution of this protein (**b**), mainly in the cell cycle (**c**) and apoptosis pathways (**d**)

BRCA1 is located on chromosome 17 and consists of 22 coding exons, which exon 11 is considered as the largest one, encoding over 60% of total 1863 amino acids encoded by the BRCA1 gene (Fig. 4a). BRCA1 gene is responsible for the translation of full-length BRCA1 protein [26] and over 4000 genetic variants of this gene have been functionally identified [27]. The mature and full-length of BRCA1 protein is located in the nucleus and consists of several functional domains, including N-terminal zinc-binding RING finger domain (amino acids #10-109), BRCA1 C-terminal (BRCT) domain (amino acids #1640-1729, #1760-1821), two nuclear localization signals (NLS), a coiled-coiled domain (amino acids #1367-1437), and Serine– Glutamine (SQ) cluster (amino acids #1280-1524) [28] (Fig. 4a).

The zinc-binding RING finger motif is the main functional part of BRCA1 and it is important for E3-ubiquitin ligase activity of this protein. This motif heterodimerizes with BRCA1-Associated RING Domain 1 (BARD1) and the tandem BRCT domain, resulting in various protein-protein interactions through binding to phosphorylated serine [29].

BRCT domain appears as a tandem repeat in BRCA1 gene and it is responsible for the phosphoprotein interactions between BRCA1 and other phosphorylated proteins involved in DNA damage response, such as CtIP, BRIP1, and Abraxas [30, 31]. Shakya et.al demonstrated that the BRCT domain is critical for the genome stability function of BRCA1, and S1598F point mutation in this domain disrupts the genomic stability function of BRCA1 and causes tumors development like when BRCA1 is completely deactivated [32]. Moreover, a recent study revealed that the interaction between this domain and mTORC2 impairs Akt activation, which is necessary for the proliferation of cancer cells [33]. Several BRCT-associated mutations have been recognized, which are able to disturb different functions of this protein, including damage foci localization, protein stability, and resection-dependent Homology directed repair (HDR) and Single Strand Annealing (SSA) [34–37].

The region between the RING and BRCT domains is called the central region of BRCA1. The central region was not studied properly as two previous domains [38]. Recently Lin et.al reported this region contains nine highly-conserved motifs, which are necessary for DNA repair activity of BRCA1 and the deletion of these motifs could decrease cell viability following cisplatin treatment [39].

The transportation of BCRA1 from the cytosol to the nucleus is controlled by two NLS domains, which are recognized by the importin-α machinery. Mutation in the NLSs domain causes accumulation of BRCA1 in the cytosol and reduce the tumor suppressor activity of this protein [40].

The coiled-coil domain of BRCA1 is located in exons 11-13 of BRCA1 and interacts with the coiled-coil domain of PALB2 during the HR DNA repair system [41, 42]. The SQ cluster also contributes to HR and contains several serine and threonine residues that can be phosphorylated by ATM and ATR [43, 44].

### Function

Several functions have been attributed to BRCA1 protein, including transcription-coupled repair, regulation of transcription, remodeling of chromatin, apoptosis, and ligation of ubiquitin [44] (Fig. 4b). However, the well-known function of BRCA1, acting as a tumor suppressor, is related to the role of BRCA1 in promoting genomic stability [45, 46]. In order to regulate genomic stability, BRCA1 contributes to DNA repairing pathways, participates in DNA damage-induced cell cycle checkpoint mechanisms (Fig. 4c), and induce apoptosis cascade activation (Fig. 4d) [45]. Moreover, recent evidence demonstrated BRCA1 contribution to genomic stability maintenance is associated with the prevention of tandem duplication [47] and RNA-DNA hybrid (R loop) processing [48].

### DNA repair

Several studies have reported that cells with defective BRCA1 gene are hypersensitive to DNA damaging agents, such as IR, UV, and alkylating agents. These defective BRCA1-cells fail to repair DNA damages properly, indicating the essential role of BRCA1 in the DNA repair system [9]. As discussed earlier, NHEJ and HR are two main DSB repairing pathways in every organism. BRCA1 influences the cellular choice to proceed toward NHEJ or HR pathways to repair the damages in DNA.

#### Role of BRCA1 in HR

Many studies demonstrated the direct role of BRCA1 in the HR pathway, as BRCA1 deficient cells showed severe impaired HR-mediated DSB repairing (Fig. 1) [14, 49]. Following DSB in DNA, BRCA1 binds to DSB through abraxas–RAP80 macro-complex, which induces ubiquitination of histones at DNA DSBs [14]. The formation of BRCA1–abraxas–RAP80 complex is dependent on the phosphorylation of histone H2AX (γH2AX), the mediator of DNA damage checkpoint protein 1 (MDC1) and RING finger protein 8 (RNF8) by ATM [50]. Afterward, BRCA1 via cooperating with Mre11, Rad50, and Nbs1 (MRN) forms a complex with CtIP, to promote 5′-end resection in the early steps of the synthesis-dependent strand annealing (SDSA) pathway of HR [51, 52]. Although the BRCA1–CtIP complex has been shown to be critical for the HR pathway in chicken DT40 cells, another research reported this interaction is not necessary for resection-mediated DNA repair or tumor suppression in mammalian cells [53]. In the following, BRCA1 interacts with PALB2 and BRCA2 to recruit RAD51, an essential mediator in the HR repairing pathway. The formation of BRCA1- PALB2- BRCA2 complex is relying on CHK2-mediated phosphorylation of S988 on BRCA1 [54]. Lack of BRCA1 or mutation in S971, which corresponds to human S988, breaks apart the PALB2-BRCA2 complex, which leads to the abrogated HR repair process and development of mammary and endometrial tumors in exposure to DNA damaging agents [55]. The function of BRCA1 in HR is distinct from its other functions in the DDR. Cells expressing BRCA1 mutant S988A have defective HR repair pathway, although the checkpoint regulation or resistance to ionizing radiation remains intact [56].

Furthermore, a recent study found that T1394 phosphorylation residues are influential for BRCA1-PALB2 interaction and any mutation in this site can partially impair HR pathway activity [57].

The BRCA1–BACH1 complex also contributes to the HR pathway. This complex is not HR restricted and involves many DNA repair pathways, such as cell cycle checkpoint, and DNA interstrand crosslink (ICL) repair [58]. BACH1 is one of the Fanconi anemia (FA) proteins that interact with the BRCT domain of BRCA1 through phosphoserine [59]. Mutation in BACH1 or in BRCT domain that could disrupt the interaction between BRCA1 and BACH1, affect the HR pathway, delay DNA repair, and finally increase the risk of breast cancer [60, 61].

#### Role of BRCA1 in NHEJ

BRCA1 contribution to the NHEJ repair pathway has been reported in different studies; however, there are contradictory results regarding this function. The role of BRCA1 in NHEJ has been initially determined in MEFs, indicating a significantly reduced end-joining activity in BRCA1 depleted MEFs comparing to the wild-type cells [62]. Later, other studies have reported the same decreased activity of the NHEJ pathway in BRCA1-deficient HCC1937 [63], and lymphoblastoid cell lines derived from breast cancer patients [64, 65]. Further studies demonstrated BRCA1 is required for precise end-joining, as knockdown of this gene significantly reduced the ability of cells in precise DNA repair mechanisms [66]. A similar result has been obtained, when other C-NHEJ components, including Ku70, XRCC4, and Ligase IV were knocked down [66]. Moreover, a reduced level of end-joining efficiency has been reported in BRCA1Δ14– 15 and BRCA1Δ17–19 splicing variants, suggesting that these splicing variants may have a prevailing negative effect on the efficiency of C-NHEJ [67].

In contrast, some evaluations concluded that BRCA1 is not part of NHEJ pathway in BRCA-deficient HCC1973 cell lines using pulsed-field gel electrophoresis [68, 69] and further showed sporadic breast cancer cells has intact NHEJ activity in DSB repairing [70].

### Cell cycle checkpoints

Cell cycle checkpoints have a critical role in cell survival. During DNA damage, BRCA1 contributes to cell survival through activating DNA damage checkpoints occurring in G1/S, intra-S, and G2/M phases (Fig. 4c). Eventually, the activated checkpoints block the cell cycle progression in the presence of DNA damage and prohibit the cell cycle process until the damage is fully repaired.

#### Role of BRCA1 in G1/S checkpoint

In 2004 Fabbro et al. reported cells with knockdown BRCA1 failed to undergo cell cycling progression through G1/S checkpoint, indicating the important role of BRCA in this cell cycle phase [71]. The authors reported that BRCA1 mediates the phosphorylation of p53 by ATM during DNA damage and thereby, induce the expression of cyclin inhibitor p21. In their study, BRCA1 induced phosphorylation of p53 in response to both IR and UV DNA damage; however, the role of BRCA1 in G1/S arrest was merely found following IR damage. Additionally, a recent study reported that UV exposure also disrupts the G1/S cell cycle checkpoint in primary fibroblasts from individuals with a BRCA1 ^+/-^ genotype [72].

#### Role of BRCA1 in S-phase checkpoint

S-phase checkpoint is another cell cycle checkpoint, which inhibits the cell cycle progression following DNA damage. The impaired activity of S-phase checkpoints in BRCA1 deficient HCC1937 cells during DNA damage and its restoration to normal activity by functional complementation of the BRCA1 gene indicates that BRCA1 has a critical role in S-phase checkpoint activity [73].

In response to DNA damage, ATM and ATR are activated and promote the kinase activity of Chk1 and Chk2. These two checkpoint kinases regulate the Cdc25 phosphatase family and this family (A/B/C) controls cyclins and cyclin-dependent kinases’ activity during S-phase progression [19]. It seems that BRCA1 involvement in S-phase checkpoint is mediated through regulation of Chk1 kinase activity.

Moreover, activation of S-phase checkpoint is dependent on the phosphorylation of ser1387 of BRCA1 via ATM, indicating the possible role of phosphorylated BRCA1 in recruiting the other regulating components in the signal cascade [73]. Furthermore, BRCA1 might regulate the activation of ATM following DNA damage during S-phase. Studies have demonstrated that BRCA1 interacts with the MRN complex, which monitors cells for DSBs and activates ATM directly [74, 75].

Besides, a recent study suggested that in response to DNA damage, pCAF and GCN5 acylate the lysine 830 of BRCA1 to activate this protein. SIRT1, on the other hand, inhibits the activity of BRCA1 through the deacetylation of lysine 830. BRCA1 and SIRT1 form a reciprocal loop to regulate the intra-S-phase checkpoint, maintaining genome stability and, thereby preventing tumorigenesis [76].

The BRCA1–BACH1 is another complex that is involved in the S phase. This interaction can be immediately detected during S checkpoint and it is necessary for stalling replication forks due to DSBs or DNA lesions [77, 78]. Mutation in the BRCT domain disturbs the proper connection between BRCA1 and BACH1, which results in delayed entry into the S phase of the cell cycle, defective DNA repair, and breast cancer development [60].

#### Role of BRCA1 in G2/M checkpoint

Similar to G1/S and S-phase checkpoints, G2/M checkpoint is also activated in case of DNA damage, arresting the cell cycle process, and cell division.

It has been reported that intact BRCA1 is essential for both initial and the maintenance of the G2/M checkpoint function, while BRCA2 and PALB2 are only responsible for maintaining the cell arrest [79]. Following DNA damage, abraxas–RAP80 macro-complex controls the recruitment of DNA repair proteins like BRCA1 to the sites of DNA damage and BRCA1–abraxas–RAP80 complex activates the G2/M phase cell-cycle checkpoints and cause CHK1 phosphorylation later [80]. In addition, CtIP/BRCA1 only exists in the G2 phase and has been shown to be critically involved in the G2/M transition phase checkpoint activation and CHK1 phosphorylation in response to the DNA damage [81, 82]. However, damage-induced G2 accumulation checkpoint is controlled by BRCA1–BACH1 complex, not CtIP/BRCA 1[82]

### Apoptosis

Various studies revealed the consequential role of BRCA1 in inducing apoptosis through different mechanisms (Fig. 4d). 1) BRCA1 is known to be a nuclear-cytoplasmic shuttling protein and BARD1 is responsible for transporting BRCA1 to nuclear. The BRCA1-depended apoptosis occurs when the BRCA1-BARD1 complex is disrupted and BRCA1 accumulate in the cytoplasm [83, 84]. 2) BRCA1 also mediates apoptosis through regulating the p53 inducible gene 3 (PIG3) expression [85]. PIG3 is a downstream protein of p53 and it is involved in the p53-dependent apoptosis pathway. Zhang et al. demonstrated the significant association between PIG3/BRCA1 expression and better survival of breast cancer patients [85]. 3) A correlation between BRCA1 and impaired tumor necrosis factor (TNF)-α production have been reported, which is an apoptotic inducer factor [86]. Moreover, Natriuretic peptide receptor 3 suppresses cytoplasmic BRCA1 and TNF-α and protects the cardiomyocytes from cell death [87]. 4) X-linked inhibitor of apoptosis (XIAP)-associated factor 1 (XAF1) is a tumor suppressor protein that interacts with BRCA1 and makes BRCA1 bind ERα and BRCA1-mediated K48 polyubiquitination of ERα, and finally induce BRCA1-mediated apoptosis [88]. 5) BRCA1 stimulates apoptosis through binding to the inositol 1,4,5-trisphosphate receptors (IP3R), resulting in excessive calcium release and cell death [89]. IP3R acts as a calcium channel and is activated by inositol trisphosphate. BRCA1 binds to IP3R, increase the sensitivity of this receptor to its ligand, IP3, and subsequently increases IP3R-mediated apoptotic calcium release [89]. 6) BRAC1 expression could induce apoptosis in breast cancer cell lines, in response to some stress stimuli, such as serum deprivation. This apoptotic pathway is independent of p53 function and proceeds through -Ras/MEKK4/JNK and Fas-dependent signaling pathway and activation of caspase 8 [90]. 7) Cytoplasmic BRCA1 activates Growth Arrest and DNA Damage 45 **(**GADD45) sequences in a p53-independent manner leading to cell death. GADD45 is a DNA damage-responsive gene and function in DNA repair, cell cycle checkpoint, and apoptosis pathways. BRCA1 could either activate GADD45 through interaction with Oct-1 and CAAT motifs of this gene [91] or suppress GADD45 through its interaction with a novel zinc finger protein, ZBRK1 [92]. In response to the DNA damage, BRCA1 induces the p53- independent expression of GADD45 and subsequently activates the JNK/SAPK (c-Jun N-terminal kinase/stress-activated protein kinase) pathway of cellular apoptosis [93].

## BRCA2

### Frequency

BRCA2 is another highly penetrant genes involved in hereditary breast cancer susceptibility. BRCA2 mutation increases the risk of breast cancer by 45-85% and ovarian cancer by 11-23% in the women population [94]. In addition, BRCA2 mutation has been found in 10% of pancreatic cancers studied, associated with 10-fold raised risk [95].

Unlike BRCA1 mutation carriers, the pathological feature of breast cancer patients with a BRCA2 mutation is usually similar to sporadic breast cancer. Although BRCA1 is known to be TNBC, no significant correlation between BRCA2 mutation and TNBC has been reported [96].

### Gene structure

BRCA2 was discovered in 1995 [97]. This large gene contains 27 exons, which the most predominant mutations occur in the exon 10 and exon 11 in the form of insertion and deletion, resulting in several premature stop codon ending and missense mutations [98].

**Fig. 5:**
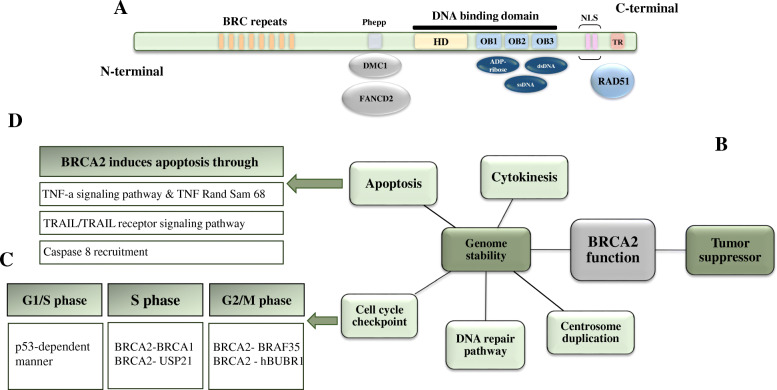
The gene structure of BRCA2 (**a**) and the overall contribution of this protein (**b**), mainly in the cell cycle (**c**) and apoptosis pathways (**d**)

BRCA2 gene encodes 3418 amino acids for different functional domains (Fig. 5a). The N terminal region of BRCA2 contains eight BRC repeats (amino acids #1009-2082) with approximately 1000 amino acids. Although the function of the N-terminal region is not clear yet, it has been reported that BRC repeats in this region are responsible for protein-protein interaction, especially between BRCA2 and RAD51. The c-terminal region of BRCA2 contains BRCA2 DNA-binding domain (amino acids #2478-3185), which comprises a helical domain (HD), three oligonucleotide/oligosaccharide-binding (OB) folds and a Tower domain (T). The helical domain encodes 190 amino acids and the three OB domains named as OB1, OB2, and OB3 contain approximately 110 amino acids. The OB domains are responsible for the high affinity of BRCA2 to ssDNA and dsDNA damage, and poly (ADP-Ribose) [98, 99]. Moreover, there is a phenylalanine-proline-proline (PhePP) motif in the C-terminal region (amino acids #2386–2411), beside the DNA-binding domain. PhePP interacts with DMC1 and FANCD2 thorough meiosis [100]. There are two NLS motifs in the c–terminal of BRCA2 (amino acids #3263-3269, #3381-3385), which are required for transferring BRCA2 to the nucleus.

### Function

BRCA2 participates in many biological activities. This protein mainly acts as a tumor suppressor gene and prevents cells from uncontrolled dividing and growth via regulation of DNA repair, cell cycle, and cell death pathways (Fig. 5b).

### DNA repair

Similar to BRCA1, BRCA2 plays a critical role in the DNA repair system. The BRCA2 deficient cells demonstrated genomic instability and caused mouse embryonic lethality. Moreover, these cells are hypersensitive to DNA damaging agents and fail to repair DNA damages properly.

#### Role of BRCA2 in HR

BRCA2 contribution in the DNA repair system is mainly through regulating the HR pathway [101] (Fig. 1). Yeast two-hybrid methodology provided the first evidence that BRCA2 is one of the crucial constituents of HR-mediated DSB repair [102]. Bradley et al. demonstrated the BRCA2-associated HR repair pathway is mediated by the interaction of BRCA2 and RAD51 [102]. RAD51 interacts with 300 residues of C-terminal region of BRCA2 and TR2 domain in C-terminal region stabilized the RAD51 nucleofilaments, especially in response to nucleotide depletion after treatment with a potent ribonucleotide reductase inhibitor [103]. Deletion of C-terminal region of BRCA2 or mutants like BRCA2 6174delT and 6158insT impair the RAD51- binding activity of this domain, diminish the RAD51 recruitment to the damage site, and thereby increase the risk of tumor incidence in mice and early onset of breast and ovarian cancers in human [104, 105]. Furthermore, BRCA2 deficient cells are more sensitive to DNA damages agents, such as poly (ADP-ribose) polymerase (PARP) inhibitors or radiation due to defective HR repair pathway [106].

RAD51 also interacts with BRC motifs of BRCA2 [107]. Point mutations in BRC motifs, especially those associated with familial early-onset cancer, significantly disturb the interaction between BRCA2 and RAD51. Overexpression of BRC motifs interrupts the formation of subnuclear foci during DNA damage and increases the sensitivity of cells to the ionizing radiation [107, 108]. The affinities of BRC motifs to RAD51 protein are varied. BRC1 is critical for the interaction between BRAC2 to RAD5; however, BRC4 has a threefold stronger ability to RAD51 compered to BRC1. G1529R mutation, which belongs to the BRC4 region, is significantly associated with the risk of familial breast cancer. BRC5 and BRC6 are not required for this interaction [109].

Normally, Cyclin A-CDK2 (or cyclin B-CDK1) phosphorylate Se3291 in the C-terminal region of BRCA2 and inhibit the RAD51 binding activity of this domain and consequently, suppress HR pathway [110]. Nevertheless, following DNA damage, the phosphorylation is halted and RAD51 is recruited to the BRCA2-containing DNA repair foci. Cyclin D1 interferes with the phosphorylation of Ser3291 by A-CDK2 and fascinated the recruitment of RAD51 to BRCA2 [111]. Overexpression of cyclin D1 has been reported in several cancer types, mainly familial breast cancer [112, 113]. Moreover, polo-like kinase 1 (Plk) improves the RAD51 recruitment and accumulation at the DNA damage site. Plk is a proto-oncogene that phosphorylates RAD51 at Serin 14 and BRCA2 facilitates this process. The Plk1 phosphorylates Rad51 at T14 by CK2, which facilitates Rad51 binding to Nbs1, and finally, increase the recruitment and accumulation of RAD51 and promote HR [114]. Furthermore, Ubiquitin-specific protease 21 (USP21) enhances the efficiency of interaction between BRCA2 and RAD51at the DNA damage site through deubiquitylating and stabilization of BRCA2. Deactivation of USP21 reduces the HR activity and increases the DNA damage frequency [115].

Although BRCA2 involvement in HR pathways is principally dedicated to RAD51 binding, additional protein-protein interactions are also involved. The BRC repeat in the N-terminal region of BRCA2 interacts with PALB2/FANCN, which physically links BRCA1 to BRCA2 in a cell cycle-dependent manner. Mutation in either BRCA2 or PALB2 is associated with reducing the ability of cells in HR repairing and accordingly, increasing the risk of breast cancer [116].

#### Role of BRCA2 in NHEJ

Although BRCA1 is involved in both HR and NHEJ repair systems, there is no strong evidence for the contribution of BRCA2 in NEHJ. Several studies reported that BRCA2 has no effect on NHEJ.

### Cell cycle checkpoints

The function of BRCA2 in controlling the cell cycle checkpoints is less studied compared to the BRCA1 (Fig. 5c). Few researchers demonstrated that truncated BRCA2 cells fail to block cell-cycle transitions during DNA damage and induce enhanced susceptibility to breast cancer, although its direct effect on cell cycle arrest is controversial and it seems the protein might cause cell cycle arrest as part of its main function in DNA repair mechanism.

#### Role of BRCA2 in G1/S checkpoint

It is not clear whether BRCA2 directly patriciates in the G1/S checkpoint. However, a recent study reported that defective BRCA2 stimulates replication stress, which causes DNA damage and G1 arrest in a p53-dependent manner. The author showed that the p53 level was increased in BRCA2 deficient cells and the G1 cell population was reduced when p53 was abrogated [106].

#### Role of BRCA2 in S-phase checkpoint

For the first time, Zwet et al demonstrated that transfecting the Chinese hamster cell V-C8 with human chromosome 13, which contains BRCA2 gene, or mouse BRCA2 cDNA, could rescue the RDS phenotype of V-C8 cells [117]. This finding showed that BRCA2 involved in S-phase, although the molecular mechanism behind was not well determined. It was speculated that BRCA2 works with BRCA1 to control this cell cycle checkpoint [118]. A recent observation found the specific expression of BRCA2 in S-phase and its important role for genome maintenance of S cells population, which directly mediates the replication stress, a hallmark of pre-cancerous lesions. BRCA2 expression in the S phase is stabilized by USP21, as USP21 loss reduces the expression of BRCA2 in this cell cycle stage [115].

#### Role of BRCA2 in G2/M checkpoint

Early studies reported that BRCA2 deficient mice have intact G2/M in response to DNA damage, seems that BRCA2 doesn’t control this cell cycle checkpoint. However, further studies demonstrated that BRCA2 deficient mice had defective spindle assembly checkpoint, acquired mutations in the components of the mitotic checkpoint, such as p53, Bubl, and Mad3L, and had defective mitotic checkpoints, all providing evidence for the role of BRCA2 in G2/M regulation [119, 120]. In contrary to BRCA1 which is involved in both initial and the maintenance of G2/M checkpoint, BRCA2 appears to be more important for the maintenance of this cell cycle [79, 121]. Following ionizing radiation, the BRCA2 knockdown cells showed G2 checkpoint arrest; however, over time, the cells overcame this checkpoint and entered mitosis, suggesting that BRCA2 is required for the G2 maintenance [79, 121]. BRCA2 mediates its function by interacting with BRCA2-associated factor 35 (BRAF35), which is a novel protein that binds to cruciform DNA. The nuclear staining revealed the colocalization of both BRAF35 and BRCA2 on mitotic chromosomes, which was concurrent with the phosphorylation of serine 28 (Ser-28) of histone H3 [122]. Furthermore, the antagonistic antibodies against either BRCA2 or BRAF35 delayed metaphase progression [122]. Moreover, Futamura et al. showed the interaction between BRCA2 and hBUBR1. hBUBR1 is a homolog of S. cerevisiae mitotic checkpoint protein BUB1 and phosphorylate BRCA2 [123].

### Apoptosis

There is sparse evidence about the direct role of BRCA2 in the induction of apoptosis (Fig. 5d). BRCA2 deficient mice showed defective cellular proliferation and died in utero [124] Moreover, transfecting Capan-1 cells, which expresses only a COOH-terminal truncated BRCA2 inhibited tumor growth in animal models and negatively regulated cell proliferation [125]. Further studies nominated TNF and TRAIL-R signaling pathways as potential pathways behind this phenomenon. Anne M. Heijink and his college performed a genome-wide functional genetic screen and identified the gene mutations that prevented cell death in BRCA2 siRNA silenced cells. They further validated their data in multiple BRCA2 deactivated breast- and leukemic cell lines and reported that deactivation of BRCA2 induces apoptosis through TNFα signaling pathway in these cell lines via downregulation of TNF receptor 1 (TNF-R1) or its downstream signaling component Sam68 [126]. In addition, another new study revealed that BRCA2 induces cell apoptosis through the TRAIL/TRAIL receptor signaling pathway and caspase 8 recruitment, apart from other functions of BRCA2 in cell cycle arrest and DNA repair [127]. However, inconsistent result was reported from a clinical study, reporting no significant difference between cellular proliferation and apoptosis between hereditary (with germline BRCA mutations) and sporadic (without BRCA mutation) ovarian tumors [128].

## Radiosensitivity assays in Breast Cancer

### Micronucleus assay

Micronuclei (MNi) acts as a biomarker for chromosome damage or entire chromosome loss. Therefore, in vitro micronucleus (MN) assay was designed to detect the genotoxic damage in the cells by scoring the presence of MNi. This test is faster than the chromosome aberration test as the population cells are in the interphase and the scoring system could be done in automation rather than manually [129].

The radiosensitivity capacity of the cells in different cell cycles is not similar. In the G0 MN assay, blood is irradiated, then cultured in the presence of phytohemagglutinin (PHA), resulting in the irradiation of T lymphocytes in the G0 phase. G0-based assays have the precondition that all lymphocytes are in the same cell cycle with G0-radiosensitivity. In contrast, in G2 MN assay cells are treated with mitogen PHA before irradiation. PHA stimulates T lymphocyte division and provides a population of cycling lymphocytes (G1, S1, G2, and M phase) after 3 days of incubation when the blood culture is irradiated [130]. In general, MNi is detectable in dividing eukaryote cells only. This technique has been further modified by Fenech and Morely, called cytokinesis-block MN (CBMN), in order to score DNA damaged in a once-divided binucleated cell, which are the cells that can express MNi. In the CBMN technique, cells are treated with cytochalasin B, which is an inhibitor of cytokinesis in cell division and the visualized binucleated cell are an indicator of cell that completed one nuclear division [131].

Nine studies compared the RS of breast cancer patients with control individuals (Table 1). The majority of studies (77.8%) reported that radiation-induced frequency of micronucleus was significantly higher in breast cancer group in comparison to control [5, 10, 11, 133, 135, 136, 145]. In contrast, Djuzenova et al. determined no significant difference between the level of MNi in breast cancer patients and healthy participants using G2 micronucleus test [12] and Francies et al. reported breast cancer patients with luminal are more radiosensitive compared to healthy control, while no difference between those with triple-negative breast cancer and healthy control has been detected [132].

**Table 1:** The radiosensitivity level of breast cancer patients, BRCA1/2 mutation carriers and breast cancer patients with radiotherapy complication, using micronucleus assay

Reference	Marker	Sample size	Cell type	Radiation dose	Result
**Breast cancer**					
Francies et al. 2019 [132]	G2 MN assay	1. BC cases (34) A. TNBC (17) B. Luminal (17) 2. Healthy controls (17)	Lymphocyte	2 and 4 Gy X-rays	**Spontaneous RS** TNBC ↔ Healthy controls Luminal ↔ Healthy controls **RS after radiation** **2-Gy X-rays** TNBC ↔ Healthy controls Luminal ↔ Healthy controls **4-Gy X-rays** TNBC ↔ Healthy controls Luminal > Healthy controls
Francies et al. 2019 [132]	G0 MN assay	1. BC cases (83) A. TNBC (17) B. Luminal (66) 2. Healthy controls (90)	Lymphocyte	2 and 4 Gy X-rays	**Spontaneous RS** BC cases > Healthy controls TNBC > Healthy controls Luminal > Healthy controls **RS after radiation** **2-Gy X-rays** BC cases > Healthy controls TNBC ↔ Healthy controls Luminal > Healthy controls **4-Gy X-rays** BC cases > Healthy controls TNBC ↔ Healthy controls Luminal > Healthy controls
Lou et al. 2008 [133]	G0 MN assay	1. BC cases (25) 2. Healthy controls (25)	Lymphocyte	3-Gy X-rays	**Spontaneous RS** BC case > Healthy controls **RS after radiation** BC case > Healthy controls
Djuzenova et al. 2006 [12]	G2 MN assay	1. BC cases (50) 2. Healthy controls (16)	PBMC	1, 2, 3 and 4 Gy X-rays	**Spontaneous RS** BC cases ↔ Healthy controls **Slope of MN induction** BC cases ↔ Healthy controls
Varga et al (2007) [11]	G0 MN assay	1. BC cases (91) 2. Healthy controls (96)	Lymphocyte	2 Gy γ-rays	**Spontaneous RS** BC cases > Healthy controls **RS after radiation** BC cases > Healthy controls
Mozdarani et al. 2005 [5]	G0 MN assay	1. BC cases (50) 2. Healthy controls (40)	Lymphocyte	3 Gy γ-rays	**Spontaneous RS** BC cases > Healthy controls **RS after radiation** BC cases > Healthy controls
Ban et al. 2004 [10]	G0 MN assay	1. BC cases (130) 2. Healthy controls (48).	Lymphocyte	2 Gy X-rays	**Spontaneous RS** BC cases > Healthy controls **RS after radiation** BC cases > Healthy controls
Barber et al. (2000) [134]	G2 MN assay	1. BC cases (11) 2. First degree relative of BC cases (22) 2. Healthy controls (68)	Lymphocyte	3.5 Gy γ-rays	**Spontaneous RS** BC cases > Healthy controls Relative of BC cases > Healthy controls
Scott et al. 1999 [135]	G0 MN assay	1. BC cases (130) 2. Healthy controls (68)	Lymphocyte	3.5 Gy γ-rays	**Spontaneous RS** BC cases ↔ Healthy controls **RS after radiation** BC cases > Healthy controls
Scott et al. 1998 [136]	G0 MN assay	1. BC cases (39) 2. Healthy controls (42)	Lymphocyte	**HDR** 3.5 Gy γ-rays (dose rate 1.0 Gy min-1) **LDR** 3.5 Gy γ-rays (dose rate 0.15 Gy min-1)	**Spontaneous RS** BC cases > Healthy controls **HDR** **RS after radiation** BC cases > Healthy controls **LDR** **RS after radiation** BC cases > Healthy controls
**BRCA1/2 mutation**					
Baert et al. 2017 [137]	G2 MN assay	1. Healthy BRCA2 mutation carriers (18) 2. Non-carrier subjects from BRCA1/2 families (17) 3. Healthy controls W/O family history of BC (18)	Lymphocyte	2 Gy γ-rays	**Spontaneous RS** No difference across all groups **RS after radiation** Healthy BRCA2 mutation carrier > Healthy controls Healthy BRCA2 mutation carrier ↔ Non-carrier subjects from BRCA1/2 families
Baert et al. 2016 [130]	G2 MN assay	1. Healthy BRCA1 mutations carriers (18) 2. Healthy controls W/O family history of BC (20)	Lymphocyte	2 Gy γ-rays	**Spontaneous RS** Healthy BRCA1 mutation carriers ↔ Healthy controls **RS after radiation** Healthy BRCA1 mutation carriers > Healthy controls
Gutierrez-Enriquez et al. 2011 [138]	G2 MN assay	1. 21 BRCA1 carriers (12 BC and 9 Healthy) 2. 24 BRCA2 carriers (13 BC and 11 Healthy) 3. Familial BC cases W/O BRCA1/2 mutation (15) 4. 16 healthy controls W/O familial history of BC (5 BC and 11 Healthy)	Lymphocyte	Mitomycin C	**RS after radiation** BRCA1 carriers ↔ Healthy controls BRCA2 carriers > Healthy controls BRCA2 carriers > BRCA1 carriers
Kotsopoulos et al. 2007 [13]	G2 MN assay	1. Healthy BRCA1 mutation carriers (25) 2. Non-carrier subjects from BRCA1 families (25)	Lymphocyte	2 Gy γ-rays	**Spontaneous RS** Healthy BRCA1 mutation carriers ↔ Non-carrier subjects **RS after radiation** Healthy BRCA1 mutation carriers ↔ Non-carrier subjects
Varga et al (2007) [11]	G0 MN assay	1. BC cases (85) 2. BC cases with BRCA1 mutation (6) 3. Healthy controls (96)	Lymphocyte	2 Gy γ-rays	**Spontaneous RS** BC with BRCA1 mutation ↔ Healthy controls **RS after radiation** BC with BRCA1 mutation > Healthy controls
Baeyens et al (2002) [139]	G0 MN assay	1. BC cases with BRCA1/2 mutation (20) A. BRCA1(11) B. BRCA2 (9) 2. Healthy relative with BRCA mutation (12) A. BRCA1 (6) B. BRCA2 (6) 3. Healthy relative W/O BRCA mutation (10) A. of BRCA1 (5) B. of BRCA2 (5) 4. BC cases W/O BRCA mutation (78) 5. Healthy controls (58)	Lymphocyte	**HDR** 2 Gy γ-rays and 3.5 Gy γ-rays (dose rate 1.0 Gy min-1) **LDR** 3.5 Gy γ-rays (dose rate 4 mGy min-1)	**Spontaneous RS** BC cases > Healthy controls BC with BRCA1/2 mutation ↔ BC W/O BRCA1/2 mutation Healthy relative with and W/O BRCA1/2 mutation ↔ Healthy controls **HDR & LDR** **RS after radiation** BC with BRCA1/2 mutation > Healthy controls BC with BRCA1/2 mutation ↔ BC W/O BRCA1/2 mutation Healthy relative with BRCA1/2 mutation ↔ Healthy relative W/O BRCA1/2 mutation ↔ Healthy controls
Trenz et al (2003) [140]	G2 MN assay	1. Healthy BRCA1 mutation carrier (13) 2. Healthy controls W/O familial history of cancer (13)	Lymphocyte	2 Gy γ-rays	**Spontaneous RS** Healthy BRCA1 mutation carriers ↔ Healthy controls **RS after radiation** Healthy BRCA1 mutation carriers > Healthy controls
Trenz et al (2002) [141]	G2 MN assay	1. Healthy BRCA1 mutation carrier (10) 2. Healthy BRCA2 mutation carrier (9) 3. Healthy controls W/O familial history of cancer (14)	Lymphocyte	2 Gy γ-rays	**Spontaneous RS** Healthy BRCA1 mutation carriers ↔ BRCA2 mutation carriers **RS after radiation** Healthy BRCA1 mutation carriers ↔ BRCA2 mutation carriers Healthy BRCA1 mutation carriers > Healthy controls Healthy BRCA2 mutation carriers > Healthy controls
Rothfus et al. (2000) [142]	G2 MN assay	1. Healthy BRCA1 mutation carriers (12) 2. Non-carrier subjects from BRCA1 families (10) 3. Healthy controls W/O history of cancer (17)	Lymphocyte	2 Gy γ-rays	**RS after radiation** Healthy BRCA1 mutation carriers> Non-carrier subjects from BRCA1 families Non-carrier subjects from BRCA1 families ↔ Healthy controls
**Radiotherapy complications**					
Finnon et al. 2012 [143]	G0 MN assay	1. BC cases with ASR (31) 2. BC cases with mild late adverse reaction (28)	PBMC	3.5 Gy X-rays	**RS after radiation** Marked reaction ↔ Mild late adverse reaction
Djuzenova et al. 2006 [12]	G2 MN assay	1. BC cases with ASR (9) 2. BC cases (50) 3. Healthy controls (16)	PBMC	1, 2, 3 and 4 Gy X-rays	**Spontaneous RS** BC with ASR > Healthy controls BC with ASR > BC cases **Slope of MN induction** BC with ASR > Healthy controls BC with ASR > BC cases
Taghavi-Dehaghani et al. 2005 [144]	G0 MN assay	BC cases with early reactions (15) BC cases with late reactions (11)	Lymphocyte	4 Gy γ-rays	**Spontaneous RS** Early reactions ↔ Late reactions **RS after radiation** Early reactions > Late reactions
Barber et al. 2000 [134]	G0 MN assay	**HDR** ASR before radiotherapy (116) Late reactions, 8-14 years after radiotherapy (47) **LDR** ASR before radiotherapy (73) Late reactions, 8-14 years after radiotherapy (26)	Lymphocyte	**HDR** 3.5 Gy γ-rays (dose rate 1.0 Gy min-1) **LDR** 3.5 Gy γ-rays (dose rate 0.15 Gy min-1)	**HDR** **RS after radiation** Acute reactions before radiotherapy ↔ 8-14 years after radiotherapy **LDR** **RS after radiation** Acute reactions before radiotherapy ↔ 8-14 years after radiotherapy

*RS* radiosensitivity, *MN* micronucleus, *BC* breast cancer, *PBMC* peripheral blood mononuclear cell, *HDR* high dose rate, *LDR* low dose rate, *CBMN* cytokinesis-block micronucleus, *ASR* adverse early skin reaction, *TNBC* triple negative breast cancer, *W/O* without, Gr: gray

↔: no significant differences at the radiosensitivity level, >: significant higher level of radiosensitivity

Almost half of the studies which compared DNA repair capacity of healthy BRCA1 mutation carrier with non-carrier controls have reported no significant results [13, 138] while others reported monoallelic BRCA1 or BRCA2 mutations are associated with an enhanced radiosensitivity [11, 130, 137, 138, 140, 141].

Although Rothfus et al. have suggested the MN test as a screening test for carriers of a BRCA1 mutation in breast cancer families, others failed to get this result [142]. It seems this approach is not likely to be useful for identification of BRCA carriers from non-carrier with familial history of breast cancer [130, 137, 140–142].

To determine whether MN assay is capable to predict breast cancer patients with advanced radiotherapy complications, two studies reported that the level of MNi in cancer patients with an early adverse skin reaction was significantly higher than the unselected breast cancer group [12] and late reaction [144]. However, Finnon et al. found no evidence of a differential response between breast cancer patients with marked or mild late adverse responses to adjuvant breast radiotherapy [143]. Barber et al. also concluded no trends towards increased chromosomal RS between acute and late reactions following radiotherapy [134].

### G2/0 chromosomal assay

After the MN assay, the chromosomal radiosensitivity assay is a cell-cycle-based technique that has been used extensively to investigate the association between human chromosomal RS and susceptibility to cancer or radiotherapy outcome. G2 assay most often applied on PHA-stimulated peripheral blood T-lymphocytes although it can measure the chromatid aberrations in any dividing population of cells, such as skin fibroblasts. In this technique, cells are exposed to invitro-radiation during the G2 phase of the cell cycle. The chromatid gaps and breaks can be observed in cells that progressed to metaphase. Briefly, cells are cultured for 71–72 h before irradiation. After 30 minutes of recovery, cells are treated with colcemid for 1 h. The cells observed at metaphase are those that were radiated in the G2 phase of the cell cycle [146].

In all reported results, G2 assay able to detect the RS differences in healthy donor and breast cancer patients [135, 147–152] (Table 2). About BRCA1/2 carriers, Ernestos et al. [153] and Baeyens et al. [139] have reported that breast cancer patients with BRCA1 or BRCA2 mutations were not radiosensitive than healthy women carrying no mutation.

**Table 2:** The radiosensitivity level of breast cancer patients, BRCA1/2 mutation carriers and breast cancer patients with radiotherapy complication, using G0/G2 chromosomal assay

Reference	Marker	Sample size	Cell type	Radiation dose	Result
**Breast cancer**					
Ryabchenk et al. 2012 [148]	G0 and G2 chromosomal assay	1. BC cases (37)	Lymphocyte	**G0 assay**	**G0 assay & G2 assay**
		2. Healthy controls (44)		1.5 Gy	**Spontaneous RS**
				X-rays	BC cases ↔ Healthy controls
				**G2 assay**	**G0 assay & G2 assay**
				0.5 Gy	**RS after radiation**
				X-rays	BC cases > Healthy controls
Bryant et al. 2012 [151]	G2 chromosomal assay	1. BC cases (89)	Lymphocyte	0.4 Gy	**RS after radiation**
		2. Healthy controls (96)		γ-rays	BC cases > Healthy controls
Wang et al. (2012) [152]	G2 chromosomal assay	1. BC cases (515)	Lymphocyte	1.5 Gy	**non-Hispanic White**
		2. Healthy controls (402)		γ-rays	BC cases > Healthy control
					**Mexican American**
					BC cases > Healthy control
					**African Americans**
					BC cases ↔ Healthy controls
Poggioli et al. 2010 [150]	G0 & G2 chromosomal assay	1. BC cases (23)	Lymphocyte	**G2 assay**	**G0 assay & G2 assay**
		2. Healthy controls (23)		0.4 Gy	**Spontaneous RS**
				X-rays	BC cases ↔ Healthy controls
				**G0 assay**	**G0 assay & G2 assay**
				2 Gy	**RS after radiation**
				X-rays	BC cases > Healthy controls
					G2 assay could be more appropriate to define the individual RS if compare with G0 assay
Howe et al. 2005 [149]	G2 chromosomal assay	1. BC cases (27)	Lymphocyte	0.5 Gy	**RS after radiation**
		2. Healthy controls (14)		γ-rays	BC cases > Healthy controls
Riches et al. 2001 [147]	G2 chromosomal assay	1. BC cases (65)	Lymphocyte	0.4 Gy	**RS after radiation**
		2. Healthy controls (66)		γ-rays	BC cases > Healthy controls
Scott et al. 1999 [135]	G2 chromosomal assay	1. BC cases (135)	Lymphocyte	0.5 Gy	**Spontaneous RS**
		2. Healthy controls (105)		X-rays	Healthy control ↔ BC cases
					**RS after radiation**
					BC cases > Healthy controls
**BRCA1/2 mutation**					
Ernestos et al. 2010 [153]	G2 chromosomal assay	1. BC cases with BRCA1/2 mutation (15)	Lymphocyte	1 Gy	**RS after radiation**
		2. Healthy BRCA1/2 mutation carriers (5)		γ-rays	BC with BRCA1/2 mutation > Healthy controls
		3. Healthy controls W/O familial history of cancer (21)			Healthy BRCA1/2 mutation carriers > Healthy controls
Baeyens et al (2002) [139]	G2 chromosomal assay	1. BC cases with BRCA1/2 mutation (20)	Lymphocyte	0.4 Gy	**Spontaneous RS**
		A. BRCA1(11)		γ-rays	No difference across all groups
		B. BRCA2 (9)			**RS after radiation**
		2. Healthy relatives with BRCA mutation (12)			BC with BRCA1 mutation ↔ BC W/O BRCA mutation ↔ healthy controls
		A. BRCA1 (6)			BC with BRCA2 mutation > healthy controls
		B. BRCA2 (6)			Healthy relatives with and W/O a BRCA1 mutation ↔ healthy controls
		3. Healthy relatives W/O BRCA mutation (10)			
		A. of BRCA1 (5)			
		B. of BRCA2 (5)			
		4. BC cases W/O BRCA mutation (78)			
		5. Healthy controls (58)			
**Radiotherapy complications**					
Finnon et al. 2012 [143]	G2 chromosomal assay	1. BC cases with marked reaction (31)	Lymphocyte	3.5 Gy	**RS after radiation**
		2. BC cases with mild late adverse reaction (28)		X-rays	Marked reaction ↔ Mild late adverse reaction
Barber et al. 2000 [134]	G2 chromosomal assay	**HDR** Acute reactions before radiotherapy (116) Late reactions, 8-14 years after radiotherapy (47) **LDR** Acute reactions before radiotherapy (73) Late reactions, 8-14 years after radiotherapy (26)	Lymphocyte	**HDR**	**HDR**
				3.5 Gy γ-rays (dose rate 1.0 Gy min-1)	**RS after radiation**
					Acute reactions before radiotherapy ↔ 8-14 years after radiotherapy
				**LDR**	
				3.5 Gy γ-rays (dose rate 0.15 Gy min-1)	**LDR**
					**RS after radiation**
					Acute reactions before radiotherapy ↔ 8-14 years after radiotherapy

*RS* radiosensitivity, *BC* breast cancer, *HDR* high dose rate, *LDR* low dose rate, *W/O* without, *Gr* gray

↔: no significant differences at the radiosensitivity level, >: significant higher level of radiosensitivity

Regarding the radiotherapy complications, no trends towards increased chromosomal RS between acute and late adverse reactions [134] or between marked and mild late adverse reactions [143] following radiotherapy were reported.

### Comet assay

Comet Assay also called single cell gel electrophoresis (SCGE), is a sensitive and rapid technique for detecting chromosome aberration in eukaryotic cells. This technique was first introduced by Swedish researchers Östling & Johansson in 1984 [154] and modified four years later as Alkaline Comet Assay by Singh, et al. [155]. The alkaline comet assay detects a wide range of DNA damage including SSB, DSB, and alkaline- labile sites. Another most common types of comet assay is the neutral comet assay, which is more specific for detecting DSB [156].

Three Studies evaluated the RS level in breast cancer patients in comparison to healthy donors (Table 3). Two studies found no significant difference in radiation-induced DNA damage in cancer cases and healthy donors [12, 157] while LOU et al. found a significantly higher level of DNA damage in breast cancer patients [133]. Similarly, in another study, Zhang et al. found malignant breast cancer patients showed a significant upper rank of residual DNA double-strand than patients with benign breast disease in neutral comet assay [156].

**Table 3:** The radiosensitivity level of breast cancer patients, BRCA1/2 mutation carriers and breast cancer patients with radiotherapy complication, using comet assay

Reference	Marker	Sample size	Cell type	Radiation dose	Result
**Breast cancer**					
Lou et al. 2008 [133]	Comet assay	1. BC cases (25)	Lymphocyte	3-Gy	**Spontaneous RS**
		2. Healthy controls (25)		X-rays	Healthy controls ↔ BC cases
					**RS after radiation**
					BC cases > Healthy controls
Shahidi et al. 2007 [157]	Alkaline and Neutral Comet assay	1. BC cases (35)	Lymphocyte	**Alkaline**	**Alkaline & Neutral Comet assay**
		2. Healthy controls (29)		1 Gy	**Spontaneous RS**
				γ-rays	BC cases > Healthy controls
				**Neutral**	**Alkaline & Neutral Comet assay**
				2 Gy	**Initial RS**
					Alkaline & Neutral Comet assay
				γ-rays	BC cases ↔ Healthy controls
Djuzenova et al. 2006 [12]	Comet assay	1. BC cases (50)	PBMC	5 Gy	**Spontaneous RS**
		2. Healthy controls (16)		X-rays	BC cases ↔ Healthy controls
					**Initial RS**
					BC cases ↔ Healthy controls
					**Residue RS**
					BC cases ↔ Healthy controls
Zhang et al. 2006 [156]	Comet assay	1. Malignant breast tumor (14)	PBMC	0.5 Gy	**Spontaneous RS**
		2. Benign breast tumor (18)		γ-rays	Malignant ↔ Benign
					**Residue RS**
					Malignant > Benign
**BRCA1 mutation**					
Kotsopoulos et al. 2007 [13]	Comet assay	1. Healthy BRCA1 mutation carriers (25)	Lymphocyte	2 Gy	**Spontaneous RS**
		2. Non-carrier control (25)		γ-rays	Healthy BRCA1 mutation carriers ↔ Non-carrier controls
					**RS after radiation**
					Healthy BRCA1 mutation carriers ↔ Non-carrier controls
Trenz et al (2002) [141]	Comet assay	1. Healthy BRCA1 mutation carriers (5)	Lymphocyte	2 Gy	**RS after radiation**
		2. Healthy BRCA2 mutation carriers (3)		γ-rays	BRCA1 mutation carriers ↔ BRCA2
		3. Healthy controls W/O familial history of cancer (6)			mutation carriers
					BRCA1/2 mutation carriers ↔ Healthy controls
Rothfus et al. (2000) [142]	Comet assay	1. Healthy BRCA1 mutation carriers (12)	Lymphocyte	2 Gy	**RS after radiation**
		2. Non-carrier subjects from BRCA1 families (10)		γ-rays	No difference across all groups
		3. Healthy controls W/O history of cancer (17)			
**Radiotherapy complications**					
Djuzenova et al. 2006 [12]	Comet assay	1. BC cases with ASR (9)	PBMC	5 Gy	**Spontaneous RS**
		2. BC cases (50)		X-rays	BC with ASR ↔ Healthy controls
		3. Healthy controls (16)			BC with ASR ↔ BC cases
					**RS after radiation**
					BC with ASR ↔ Healthy controls
					BC with ASR ↔ BC cases
Oppitz et al. 2002 [158]	Comet assay	1. BC cases (32)	Lymphocyte & Fibroblast	**Lymphocyte**	**Lymphocyte**
		A. Lymphocyte (30)		3Gy	**RS after radiation**
		B. Fibroblast (26)		**Fibroblast**	Elevated acute reactions > Average acute reactions
				5Gy	**Fibroblast**
					**Radiosensitivity after radiation**
					Elevated acute reactions ↔ Average acute reactions

*RS* radiosensitivity, *BC* breast cancer, *PBMC* peripheral blood mononuclear cell, *W/O* without, *Gr* gray

↔: no significant differences at the radiosensitivity level, >: significant higher level of radiosensitivity

Healthy BRCA1 mutation carriers (heterozygous genotype) and non-carrier control had a similar mean tail moment at baseline, and following g-irradiation. It seems that the use of comet assay for the detection of DNA repair capacity in healthy BRCA1 mutation carriers would be limited [13].

For the predicative purpose of radiotherapy complication by comet assay, [12, 158], Oppitz et al. measured the radiosensitivity in lymphocytes, PBMC, and fibroblast of breast cancer patients and compared with the clinical acute reaction to radiotherapy. A significant association between RS level and adverse early skin reaction was found in lymphocytes cell, but not in PBMC and Fibroblast [158].

### Bio markers

In response to DSB, the histone H2 variant H2AX is phosphorylated at its carboxyl-terminus on the conserved serine 139 residues and named γ-H2AX [159]. γ -H2AX is recognized as the biomarker of DSB, which can be visualized within minutes of exposure [50]. Apart from H2AX, P53 binding protein (53BP1) is another damage sensor of DSBs [160] that is localized in damage site and mediates the recruitment of BRCA1 by methylated H3 Lys 79 and signals chromatin/DNA damage [161]. Following DNA damage, 53BP1 is rapidly phosphorylated by ATM on multiple residues such as serine 25 (Ser25) and serine 1778 (Ser1778) [162–164]. The phosphorylated 53BP1 localizes in the damage site and mediates the recruitment of BRCA 1[165–167].

Djuzenova et al. reported γ-H2AX assay may be useful for screening the radiosensitivity in breast cancer patients (Table 4). In their study the number of γ-H2AX foci was significantly higher in unselected breast cancer patients compared to healthy volunteers in both initial (0.5 Gy, 30 min) and residual (2 Gy, 24 h post-radiation) DNA damage. For 53bp1, a higher level of foci was detected in the residual DNA damage only [169]. Similarly, another study reported the correlation between immunofluorescence of γ- H2AX/53BP1 residual in breast cancer patients with healthy volunteers [168].

**Table 4:** The radiosensitivity level of breast cancer patients, BRCA1/2 mutation carriers and breast cancer patients with radiotherapy complication, using H2AX, P53bp biomarkers

Reference	Marker	Sample size	Cell type	Dose	Result
Chua et al. 2014 [168]	γH2AX/53BP1	1. BC cases (16)	Lymphocyte	4 Gy	**Spontaneous RS**
		2. Healthy controls (8)		X-rays	BC cases ↔ Healthy controls
					**Residual RS (24h post-radiation)**
					BC cases > Healthy controls
Djuzenova et al. 2013 [169]	γH2AX	1. BC cases (57)	PBMC	0.5 Gy & 2 Gy	**Spontaneous RS**
		2. Healthy controls (12)		X-rays	BC cases ↔ Healthy controls
					**Initial RS (0.5 Gy, 30min post-radiation)**
					BC cases > Healthy controls
					**Residual RS (2 Gy, 24h post-radiation)**
					BC cases > Healthy controls
Djuzenova et al. 2013 [169]	53BP1	1. BC cases (57)	PBMC	0.5 Gy & 2 Gy	**Spontaneous RS**
		2. Healthy controls (12)		X-rays	BC cases ↔ Healthy controls
					**Initial RS (0.5 Gy, 30min post-radiation)**
					BC cases ↔ Healthy controls
					**Residual (2 Gy, 24h post-radiation)**
					BC > Healthy controls
**BRCA1 mutation carriers**					
Kotsopoulos et al. 2007 [13]	γ -H2AX	1. Healthy BRCA1 mutation carriers	Lymphocyte	2 Gy	**Spontaneous RS**
		2. Non-carrier controls (25)		γ-rays	Healthy BRCA1 mutation carriers ↔ Non-carrier controls
					**RS after radiation**
					Healthy BRCA1 mutation carriers ↔ Non-carrier controls
**Radiotherapy complications**					
Chua et al. 2014 [168]	γH2AX/53BP1	1. BC cases with minimal late complication (8)	Lymphocyte	4 Gy	**Spontaneous RS**
				X-rays	Marked late complication ↔ Minimal late complication
		2. BC cases with marked late complication (8)			**Residual RS (24h post-radiation)**
					Marked late complication > Minimal late complication
Djuzenova et al. 2013 [169]	H2AX	1. BC cases with adverse acute skin reaction (6)	PBMC	0.5 Gy & 2 Gy	**Spontaneous RS**
				X-rays	Adverse acute skin reaction ↔ Normal skin reaction
		2. BC cases with normal skin reaction (31)			**Initial RS (0.5 Gy, 30min)**
					Adverse acute skin reaction > Normal skin reaction
					**Residual RS (2 Gy, 24h post-radiation)**
					Adverse acute skin reaction > Normal skin reaction
Finnon et al. 2012 [143]	H2AX	1. BC cases with marked adverse reaction (31)	Lymphocyte	4 Gy	**Residual RS (6 h & 24h post-radiation)**
				X-rays	Marked adverse reaction ↔
		2. BC cases with mild late adverse reaction (28)			Mild late adverse reaction
Chua et al. 2011 [170]	γH2AX/53BP1	1. BC cases with severely marked reaction (7)	Lymphocyte	0.5 and 4 Gy	**Initial RS (0.5 Gy, 30min post-radiation)**
				X-rays	Severely marked reaction ↔ Minimal skin reaction
		2. BC cases with minimal skin reaction (7)			**Residual RS (4 Gy, 24h post-radiation)**
					Severely marked reaction > Minimal skin reaction

*RS* radiosensitivity, *BC* breast cancer, *PBMC* peripheral blood mononuclear cell, *W/O* without, *Gr* gray

↔: no significant differences at the radiosensitivity level, >: significant higher level of radiosensitivity

Healthy BRCA1 mutation carriers and non-carriers showed a similar level of γ-H2AX nuclear foci after exposure to radiation, indicating γ-H2AX nuclear foci assay is not likely able to distinguish women at a high risk of hereditary breast cancer [13].

Increased chromosomal radiosensitivity, quantified by γ-H2AX/53BP [168, 170] and γ-H2AX [169] immunofluorescence microscopy were observed in breast cancer patients with an adverse acute skin reaction compared to those with normal skin reaction after radiotherapy. The controversial result appeared from Finnon et al study, reported no significant association between γ -H2AX foci number in breast cancer patients with a marked adverse reaction to adjuvant breast radiotherapy with those manifesting mild late adverse reactions [143].

### Colony forming assay

Colony formation is another technique to measure the intrinsic cellular radiosensitivity of tumors. It is based on the capability of a single cell to undergo multiple divisions and grow into a colony form. In the presence of DNA damage, cells fail to proliferate and lose their colony formation capacity, whereas those with intact DNA are able to survive during radiation, retain their reproductive ability and form visible colonies under a microscope [171].

Breast cancer patients with severe reactions to radiotherapy were more sensitive to invitro iodine radiation than healthy donors [172], but no evidence of a differential response was reported between breast cancer patients without radiotherapy complications and healthy donors [172] (Table 5). Moreover, colony-forming assay failed to detect the ionizing radiation sensitivity between breast cancer patients with elevated acute reactions and with average acute reactions [158].

**Table 5:** The radiosensitivity level of breast cancer patients, BRCA1/2 mutation carriers and breast cancer patients with radiotherapy complication, using other assays

Reference	Marker	Sample size	Cell type	Radiation dose	Result
**Breast cancer**					
Auer et al. 2014 [173]	3-color FISH	1. BC cases (67) 2. Healthy controls (62)	Lymphocyte	2 Gy	**Spontaneous RS** BC cases ↔ Healthy controls **RS after radiation** BC cases > Healthy controls
Barwell et al. 2007 [174]	Terminal restriction fragment length assay	1. BC cases (212) 2. Healthy controls (1804)	WBC & lymphocyte	1. caesium-137 source at 0.5/1 Gy, or mock 2. caesium-137 source at 4 Gy, or mock	**RS after radiation** BC cases ↔ Healthy controls
West et al. 1995 [172]	Colony formation	1. BC cases with acute complication (7) 2. BC cases with late complication (6) 3. BC cases W/O complication (8) 4. Healthy controls (20)	Lymphocyte	**HDR:** 1.55 Gy min^-1^ **LDR:** 0.0098 Gy min^-1^	**HDR** **RS after radiation** BC cases ↔ Healthy controls **LDR** **RS after radiation** BC cases ↔ Healthy controls
**Radiotherapy complications**					
Oppitz et al. 2002 [158]	Colony formation	1. BC cases with elevated acute reactions (6) 2. BC cases with average acute reactions (17)	Fibroblast	5 Gy	**Spontaneous RS** Elevated acute reactions ↔ Average acute reactions **Radiosensitivity after radiation** Elevated acute reactions ↔ Average acute reactions
West et al. 1995 [172]	Colony formation	1. BC cases with acute complication (7) 2. BC cases with late complication (6) 3. BC cases without complication (8) 4. Healthy controls (20)	Lymphocyte	**HDR:** 1.55 Gy min^-1^ **LDR:** 0.0098 Gy min^-1^	**HDR** **RS after radiation** BC with acute/late complication ↔ Healthy controls BC W/O complication ↔ Healthy controls **LDR** **RS after radiation** BC with acute/late complication > Healthy controls BC cases W/O complication ↔ Healthy controls

*RS* radiosensitivity, *BC* breast cancer, *WBC* white blood cell, *HDR* high dose rate, *LDR* low dose rate, *W/O* without, Gr: gray

↔: no significant differences at the radiosensitivity level, >: significant higher level of radiosensitivity

### Other assays

#### Telomere length assay

A telomere is a repetitive sequence structure at the end of the chromosome [175]. This specialized structure is considered as a natural DSB and acts as an inhibitor of the DSB repair pathways and DNA damage checkpoints [176].

During the division of somatic cells, the length of telomeres gradually gets shorter and this process is fascinated by various endogenous and exogenous pathogenic factors such as radiation, aging, smoking, mental stress and, etc. [177–184]. Studies showed late generation (G5–G6) mTR^−/−^ mice were more sensitive to radiation compared with G2 mTR^−/−^ mice, which were also deficient in telomerase activity but had longer telomere [185, 186].

Multiple methods have been developed to estimate the study of telomere including; Terminal Restriction Fragmentation (TRF), Polymerase Chain Reaction-based Technique (PCR), Single Telomere Length Analysis (STELA), Quantitative Fluorescence in situ Hybridization (Q-FISH).

TRF is often considered as the gold standard method to study telomere [187]; however, this technique failed to distinguish the level of chromosomal radiosensitivity between newly diagnosed breast cancer patients and healthy controls [174] (Table 5).

#### Fluorescence in situ hybridization

Fluorescence in situ hybridization (FISH), is a very highly sensitive technique that individual chromosomes are printed using a specific probe [188]. The painted chromosomes are easily visualized and the DNA damage could be scored accurately in metaphase spreads. Moreover, different types of stable DNA damage including, translocations, insertions and deletions, and unstable damage such as di centric chromosomes, rings, and acentric fragments could be differentiated [188]. Using FISH assay, Auer et al. demonstrated that breast cancer patients were significantly more sensitive compared to healthy controls [173] but not in Barwell et al.’s study [174] (Table 5).

In summary, the majority of studies confirmed the enhanced spontaneous & radiation-induced radiosensitivity of breast cancer patients compared to healthy controls (Table 6). Patients with sporadic breast cancer also had lower DNA damage capacity compared to cancer-free population, suggesting other low penetrance genes involved in DNA repair pathways, and cell cycle and apoptosis cascades, such as p53bp, ATM, BARD1, and PALb2 are involved in increased radiation susceptibility and could be a risk factor for both inherited and some sporadic breast cancer development. Therefore, evaluating the overall individual capacity of repairing DNA damages through different experimental approaches could identify the hypersensitive patients and become a marker of cancer proneness. Here we have found that MN test, G0/2 chromosomal assay, and biomarkers provided more reproducible data compared to the other assays (Table 6).

**Table 6:** Glance over the ability of different assays in distinguishing the radiosensitivity level among breast cancer patients, BRCA1/2 mutation carriers and breast cancer patients with radiotherapy complication

	BC cases vs Healthy controls	Healthy BRCA1 mutation carriers vs Healthy controls	Healthy BRCA2 mutation carriers vs Healthy controls	Healthy BRCA1/2 mutation carriers vs Non-carrier subjects from BRCA1/2 families	BC with BRCA1/2 mutation vs BC W/O BRCA1/2 mutation	BRCA2 vs BRCA1	Radiotherphy Complication
G2 MN assay	**- - +**	**+ - + +**	**+ +**	**- +**		**+ -**	**+**
G0 MN assay	**+ + + + + + +**	**-**	**-**	**-**	**-**		**- + -**
G2 chromosomal assay	**+ + + + + + +**	**+ -**	**+ +**		**-**		**- -**
G0 chromosomal assay	**+ +**						
Comet assay	**+ - - +**	**- - -**	**-**	**-**			**+ -**
H2AX, P53bp biomarkers	**+ + +**	**-**					**+ + - +**

*BC* breast cancer, *MN* micronucleus, *W/O* without

+ the technique was able to detect the more radiosensitive group

- the technique failed to detect the more radiosensitive group

BRCA1 and BRCA2 are highly penetrated genes involved in the familial breast cancer development and about 15 % of all familial breast cancer can be attributed to a mutation in these genes. Using G2 MN and G2 chromosomal assays, some studies have reported the lymphocyte of healthy BRCA1/2 mutation carriers (heterozygous genotype) are hypersensitive to invitro ionizing radiation compared to non-carriers without a history of breast cancer. BRCA1/2 mainly function in the HR pathway. Since the HR repair pathway exclusively takes place in the late S and G2 phases of the cell cycle, increased radiosensitivity in patients harbouring BRCA1/2 mutations is mostly detected when the radiation takes place in the G2 phase. However, inconsistent evidence also exists and other studies using comment assay, and H2AX biomarker failed to detect the significant differences between these two groups as well.

Limited studies compared the radiosensitivity of healthy BRCA1/2 mutation carriers and non-carriers in the BRCA families (Table 6). Although Rothfus et al. have suggested the MN test has a potential to be a screening test for carriers of a BRCA1 mutation in breast cancer families, others failed to achieve this result. It seems this approach is not likely to be useful for the identification of BRCA carriers from non-carrier with familial history of breast cancer. Developing novel radiosensitivity assays could be a promising approach in evaluating the DNA repair capacity of individuals with BRCA1/2 mutation and consider as a predictive factor for overall increased risk mainly in the relatives of breast cancer patients.

In addition, breast cancer patients with acute early reactions to radiotherapies are more radiosensitive than those with mild/no late reactions; however, inconsistent results appear among different assays (Table 6). G2 chromosomal assay failed to differentiate these differences, while most of H2AX/p53bp biomarkers seem to be able to predict those susceptible to radiotherapy complications.

Some studies have demonstrated that the presence of BRCA1/2 mutations may increase the radiotherapy complication but others not. In the reviewed population, the genetic background of breast cancer patients has not been defined; therefore, it is not possible to figure out whether radiosensitivity assays are able to screen the BRCA1-2 mutation carrier for radiotherapy complications.

## Conclusion

BRCA1 and BRCA2 are two highly penetrant genes involved in inherited breast cancer and contribute to different DNA damage pathways and cell cycle and apoptosis cascades. Breast cancer patients are more radiosensitive compared to healthy control; however, inconsistent results exist about the ability of current radiosensitive techniques in screening BRCA1/2 carriers or those susceptible to radiotherapy complications. Therefore, developing novel radiosensitivity assays could be a promising approach for pre-screening the BRCA1/2 mutation carriers and predict the overall increased risk mainly in the relatives of breast cancer patients. Moreover, it can provide more evidence about who is susceptible to manifest severe complication.

## Data Availability

All the data supporting the results are included in the article

## Abbreviations

DSBs: Double-Strand Breaks
HR: Homologous Recombinant
NHEJ: Non-Homologous End Joining
MRN: Mre11-RAD50-Nbs1
DNA-PK: DNA dependent Protein Kinases
CDK: Cyclin-Dependent Kinases
Rb: Retinoblastoma
ATR: Ataxia Telangiectasia mutated and Rad3 related kinase
TNBC: Triple-Negative Breast Cancers
ER: Estrogen Receptor
PR: Progesterone Receptor
HER2: Human Epidermal Growth Factor Receptor 2 (HER2)
CK: Cytokeratin
BRCT: BRCA1 C-terminal
NLS: Nuclear Localization Signals
SQ: Serine– Glutamine
BARD1: BRCA1-Associated RING Domain 1
HDR: Homology Directed Repair
SSA: Single Strand Annealing
PALB2: Partner and Localizer of BRCA2
γH2AX: histone H2AX
MDC1: Mediator of DNA Damage Checkpoint Protein 1
RNF8: RING Finger Protein 8
SDSA: Synthesis-Dependent Strand Annealing
ICL: Interstrand CrossLink
FA: Fanconi Anemia
MEFs: Mouse Embryonic Fibroblasts
PIG3: P53 Inducible Gene 3
TNF-α: Tumor Necrosis Factor
XIAP: X-Linked Inhibitor of Apoptosis
XAF1: X-Linked Inhibitor of Apoptosis-associated factor 1
IP3R: Inositol 1,4,5-trisphosphate Receptors
GADD45: Growth Arrest and DNA Damage45
JNK/SAPK: c-Jun N-terminal Kinase/Stress-Activated Protein Kinase
HD: Helical Domain
OB: Oligonucleotide/Oligosaccharide-Binding
PhePP: Phenylalanine-Proline-Proline
PARP: Poly (ADP-Ribose) Polymerase
Plk: Polo-like kinase 1
USP21: Ubiquitin-Specific Protease 21
BRAF35: BRCA2-associated Factor 35
Ser-28: Serine 28
TNF-R1: TNF Receptor 1
MNi: MicroNuclei
MN: MicroNucleus
PHA: PhytoHemAgglutinin
CBMN: Cytokinesis-Block MN
SCGE: Single Cell Gel Electrophoresis
PBMC: Peripheral Blood Mononuclear cell
H2AX: Histone Family member X
P53BP: P53 Binding Protein
Ser25: Serine 25
Ser1778: Serine 1778
TRF: Terminal Restriction Fragmentation
PCR: Polymerase Chain Reaction-based Technique
STELA: Single Telomere Length Analysis
Q-FISH: Quantitative Fluorescence in situ Hybridization
FISH: Fluorescence in situ Hybridization
